# Assessment of Adverse Events Using the Therapy–Disability–Neurology (TDN) Grading System in a Cohort of Aneurysmal Subarachnoid Hemorrhage Patients: A Single-Center Retrospective Cohort Study

**DOI:** 10.3390/brainsci16060599

**Published:** 2026-05-31

**Authors:** Vincens Kälin, Alexis Paul Romain Terrapon, Anna Maria Zeitlberger, Gareth Ambler, Svenja Maschke, Ahmed El-Garci, Sara Bonasia, Oliver Bozinov, Marian Christoph Neidert, Isabel Charlotte Hostettler

**Affiliations:** 1 Department of Neurosurgery, HOCH Health Ostschweiz, Cantonal Hospital St. Gallen, 9000 St. Gallen, Switzerland; 2 Department of Neurosurgery, Inselspital, Bern University Hospital, 3010 Bern, Switzerland; 3 Department of Statistical Science, University College London, London WC1E 6BT, UK; 4 Department of Pediatric Neurosurgery, Charité Universitätsmedizin Berlin, 10117 Berlin, Germany; 5 Centro Della Colonna Vertebrale, Clinica Ars Medica, Via Grumo 16, 6929 Gravesano, Switzerland; 6 Department of Neurosurgery, Klinikum Rechts der Isar, TUM School of Medicine and Health, Technical University of Munich, 80333 Munich, Germany

**Keywords:** complications, TDN grade, aneurysmal subarachnoid hemorrhage, adverse events, length of hospital stay, functional outcome

## Abstract

**Highlights:**

**What are the main findings?**

- The TDN grade significantly correlates with length of hospital stay and functional outcomes in aSAH patients treated surgically and/or endovascularly.
- Higher complication burden as captured by the TDN grade is associated with longer hospitalization and worse functional outcomes at discharge and long-term follow-up.

**What are the implications of the main findings?**

- The TDN grade can serve as a standardized prognostic tool in aSAH, extending its applicability beyond its original perioperative neurosurgical context.
- Comprehensive complication grading may support clinical decision-making, patient counseling, and resource planning in aSAH care.

**Abstract:**

Background/Objectives: Adverse events (AE) associated with neurosurgical interventions can cause neurological deficits and impaired functional outcomes. The Therapy–Disability–Neurology (TDN) grade classifies AE severity based on treatment requirements, disability, and neurological deficits, but has not been validated in aneurysmal subarachnoid hemorrhage (aSAH). We aimed to validate the TDN grade in predicting functional outcomes and length of hospital stay (LOS) in aSAH patients, treated surgically and/or endovascularly. Methods: We conducted a single-center retrospective cohort study of a prospectively collected database of aSAH patients. Patients were recruited between 2009 and 2022. The TDN grade was retrospectively applied. Primary outcome variables were functional outcomes, assessed using the Glasgow Outcome Scale (GOS, selected for comparability with prior aSAH outcome literature), at discharge and last follow-up, and LOS. Results: We included 355 patients: mean age was 57.2 (12.9 SD) and 235 (66.1%) were female. The TDN grade showed a moderate positive correlation with length of hospital stay (rho = 0.4, *p* < 0.001). Negative correlations were observed with functional outcomes at discharge (GOS: rho = −0.56, *p* < 0.0001) and at last follow-up (GOS: rho = −0.58, *p* < 0.0001). The TDN grade demonstrated good discrimination for unfavorable outcome at last follow-up (AUC = 0.82) and good discrimination for employment status (AUC = 0.71). Patients with AEs stayed 7.63 days longer on average (*p* < 0.001). Conclusions: The TDN grade predicted hospital stay and functional outcomes in aSAH patients treated surgically and/or endovascularly, demonstrating good discrimination for unfavorable outcomes and employment status. These findings extend the grade’s applicability to both treatment- and disease-related complications and support its potential utility as a standardized tool for prognostication and resource planning. Results should be interpreted in light of the single-center retrospective design and selection bias.

## 1. Introduction

Adverse events (AEs) in neurosurgery significantly impact patient outcomes through increased mortality, morbidity, length of stay, and healthcare costs [1]. With AEs occurring in up to 25% of cases, they can dramatically affect patients’ quality of life [2].

The Clavien–Dindo classification, originally developed for general surgery, is the most widely adopted AE grading system across surgical specialties [3] and has been validated in neurosurgical populations [4,5]. However, its strictly therapy-oriented design carries an important limitation: it does not account for neurological deficits, which often dominate the clinical picture in neurosurgery yet may require no specific therapy. A patient with a new postoperative hemianopia or hemiparesis managed conservatively would be graded equivalently to a transient, fully reversible event—despite a profound and often permanent functional impact. Because the central nervous system has limited regenerative capacity, this mismatch between therapeutic burden and functional consequence is particularly critical in neurosurgical patients [6]. Several authors have highlighted this gap and proposed neurosurgery-specific modifications [7], yet no widely adopted system has fully integrated the multidimensional nature of neurosurgical complications.

Our group has recently proposed a new grading system better suited to the neurological setting, the Therapy–Disability–Neurology (TDN) grade [8]. Unlike Clavien–Dindo, which relies on a single therapy-based axis, the TDN grade integrates three independent dimensions—therapeutic consequence, disability (modified Rankin Scale), and neurological deficit—preserving comparability with established systems while explicitly capturing the functional burden that defines neurosurgical outcome. Subsequent international validation demonstrated substantial reliability and broad consensus supporting its adoption as a uniform reporting standard [9].

An accurate grading tool for neurosurgical AEs could help improve patient care through better quality management, research, and outcome prediction, and needs to be validated for different diseases and types of interventions. The original TDN publication included a cohort of all neurosurgical procedures regardless of their underlying pathology. However, the postoperative/overall course (length of hospital stay (LOS), associated rate and severity of AEs, related functional outcome) of different neurosurgical diseases differ significantly and may not be comparable.

Aneurysmal subarachnoid hemorrhage (aSAH) remains a devastating form of stroke with a high burden of disease, including both mortality and morbidity [10,11,12,13,14]. The overall outcome is often poor and is influenced by several factors, including AEs related to both endovascular or surgical treatment (e.g., postoperative hematoma requiring evacuation) and the disease itself (e.g., cerebral vasospasm) [15,16,17,18]. The evaluation of the TDN grade in predicting outcome, taking into account all AEs during hospitalization, may increase the usefulness of this tool.

The aim of this study was to validate and evaluate the TDN grade for endovascular and surgical treatment of aneurysmal subarachnoid hemorrhage as predictors of length of stay (LOS) and functional outcomes measured by Glasgow Outcome Scale (GOS) at discharge and at long-term follow-up.

## 2. Patients and Methods

### 2.1. Study Design and Population

This was a single-center retrospective cohort study using a prospectively collected database. We included all patients from our center who had proven aSAH (either by CTA, MRA, or DSA). Patients were prospectively enrolled in our institution’s SAH database between 2009 and 2022; all eligible patients from the study period were included. Some of the outcome parameters and AE for TDN calculation had to be assessed retrospectively. Patients with non-aneurysmal subarachnoid hemorrhage or aSAH due to another underlying pathology, such as arterio-venous malformation, mycotic aneurysm, and others, were not included in this study. We also excluded patients who did not receive endovascular/surgical treatment for the ruptured aneurysms or for whom there was no information on the AE, and therefore no way to determine the TDN grade, or the condition at discharge (*n* = 70, see Supplemental Figure S1 for a study flow chart). For the length of stay analysis, we excluded deaths, with *n* = 305 remaining for analysis, because death during hospitalization truncates LOS in a way that does not reflect favorable recovery and would bias the correlation toward shorter stays for the most severe cases. We recognize that this exclusion may bias the correlation downward, as the most severely affected patients are removed. To explore the impact of this exclusion, we performed a sensitivity analysis including all patients (*n* = 355) by assigning deceased patients (TDN = 5) a length of stay value at the upper extreme of the observed distribution, such that they received the highest ranks in the Spearman correlation. We note that this approach imposes a strong assumption—that deceased patients would have had the longest stays—and therefore likely biases the correlation upward. For employment analysis, only patients below retirement age in Switzerland (<65 for males and <64 for females) were included (*n* = 190). For the time to last follow-up, we used the last available follow-up; data was available for 250 patients, with a median follow-up of 22 months (mean 34 months, range 0.4–140 months).

### 2.2. Assessment of the TDN Grade

The TDN grade was assessed according to the original publication [8] using the three dimensions: therapeutic consequence of an AE according to the Clavien–Dindo grading [3], disability by assessing the modified Rankin scale (mRS) resulting from an AE, and the presence of a neurological deficit. The TDN grade was applied by two evaluators (a neurosurgical resident and a senior neurosurgical consultant) working independently. Discrepancies were resolved by consensus discussion, with a third senior author acting as arbiter when needed. An AE was defined as any deviation from the expected post-hemorrhagic or postoperative clinical course, including both intervention-related complications and disease-related complications (e.g., hydrocephalus, vasospasm, delayed cerebral ischemia, infections). This deliberately broad definition explains the high overall AE incidence observed in our cohort. We assessed the TDN grade during the index hospitalization associated with AEs related to endovascular/surgical treatment—such as post-/periinterventional bleedings, infections, periinterventional dissections, thromboembolism, postoperative infarctions, anaesthesiologic complications—as well as AEs associated with the aSAH itself, e.g., elevated intracranial pressure (ICP), hydrocephalus, cerebral salt wasting, diabetes insipidus, myocardial infarction and other heart problems, ileus, urinary tract infection, pneumonia, and pulmonary embolism (See Supplemental Table S1 for a summarized list of the AEs in our cohort).

### 2.3. Outcome Variables

Our primary outcome variables were functional outcome using Glasgow Outcome Scale (GOS) at discharge and at last follow-up, and length of hospital stay (LOS). GOS was selected for its widespread use in aSAH outcome research, allowing direct comparability with prior studies. Secondary outcomes included employability at last follow-up (analyzed in patients below retirement age) and occurrence of adverse events (AEs). Planned analyses included correlation of TDN with poor functional outcome, evaluation of risk factors for AE occurrence, and assessment of differences in TDN grade severity between endovascular and surgical treatment approaches. Dichotomized functional outcome was defined by the Glasgow Outcome Scale (GOS), with GOS 1 to 3 corresponding to unfavorable and 4 to 5 to favorable functional outcomes.

### 2.4. Statistical Analysis

We present categorical variables as count percentages and continuous variables as mean with standard deviation (SD) for normally distributed data and as median with interquartile range (IQR) for non-normally distributed data. Statistical analysis was performed by a biostatistician and a neurosurgical consultant using STATA 15 (StataCorp. 2011. Stata statistical software: Release 15. College Station, TX, USA: StataCorp LP). We report this study according to the “Strengthening the Reporting of Observational Studies in Epidemiology” (STROBE) guidelines [19]. The study was approved by the ethics committee of eastern Switzerland, with the approval number EKOS 22/179.

We assessed the predictive performance of the TDN grade by evaluating its discrimination properties for unfavorable outcome (GOS ≤ 3) at last follow-up and employment status at last follow-up. Discrimination, which measures the ability to distinguish between patients with and without poor outcomes, was evaluated using receiver operating characteristic (ROC) curves with calculation of the area under the ROC curve (AUROC). For employment analysis, we excluded patients above retirement age to focus on the working-age population (*n* = 190 included in the analysis).

The relationship between TDN grades and admission scales (WFNS, Hunt & Hess, BNI, Fisher scores), as well as outcome measures and length of stay, was analyzed using Spearman correlations due to the ordinal nature of the scales. For length of stay (LOS) analysis, we excluded patients who died during hospitalization. Risk factors for AE development were assessed using univariate logistic regression. To assess whether the TDN grade was an independent predictor of unfavorable outcome (GOS ≤ 3) at last follow-up, we additionally performed multivariable logistic regression including age, sex, baseline severity (WFNS), and TDN as independent variables, with results reported as odds ratios with 95% confidence intervals. Statistical significance was set at *p* < 0.05.

## 3. Results

We included a total of 355 patients: mean age was 57.2 (12.9 SD) and 235 (66.1%) were female. Treatment modality (clipping versus endovascular treatment) did not significantly influence the TDN grade (Table 1). However, significant differences were observed between treatment groups; posterior circulation aneurysms were more frequently treated endovascularly (35% vs. 22.2%, *p* < 0.01), EVD insertion was more common in endovascular patients (77.2% vs. 59.5%, *p* < 0.01), while craniectomy was more frequent in surgical patients (13.3% vs. 7.1%, *p* = 0.048). Mortality during hospitalization was similar between the two treatment groups (12% in the surgical vs. 16.2% in the endovascular group, *p* = 0.29).

A total of 318 patients (89.6%) developed an AE during their hospital stay. Patients developing any AE were significantly older than those who did not have any AE (57.63 vs. 49.37 years of age, *p* = 0.003). There was no sex difference between patients experiencing any AEs. Logistic regression analysis identified age as a significant risk factor for AE development (OR 1.04 per year, 95% CI: 1.01–1.07, *p* = 0.004), while sex was not a significant predictor (OR 0.95, 95% CI: 0.46–1.97, *p* = 0.898, Table 2).

Univariate logistic regression analysis identified several other significant risk factors for AE development (Table 2). All initial severity scores were strong predictors: WFNS (OR 2.973, 95% CI: 1.870–4.726, *p* < 0.001), Hunt & Hess (OR 2.961, 95% CI: 1.950–4.499, *p* < 0.001), Fisher Score (OR 4.593, 95% CI: 2.512–8.395, *p* < 0.001), and BNI Score (OR 2.564, 95% CI: 1.619–4.059, *p* < 0.001). Hypertension and diabetes mellitus were not significant predictors of AE development.

In the multivariable logistic regression for unfavorable outcome (GOS ≤ 3) at last follow-up (*n* = 301 complete cases), the TDN grade remained an independent predictor (OR 5.76, 95% CI 3.69–9.01, *p* < 0.001) after adjustment for age, sex, and WFNS score (Table 3).

### 3.1. TDN Grade and Hospital Stay

**Figure 1:**
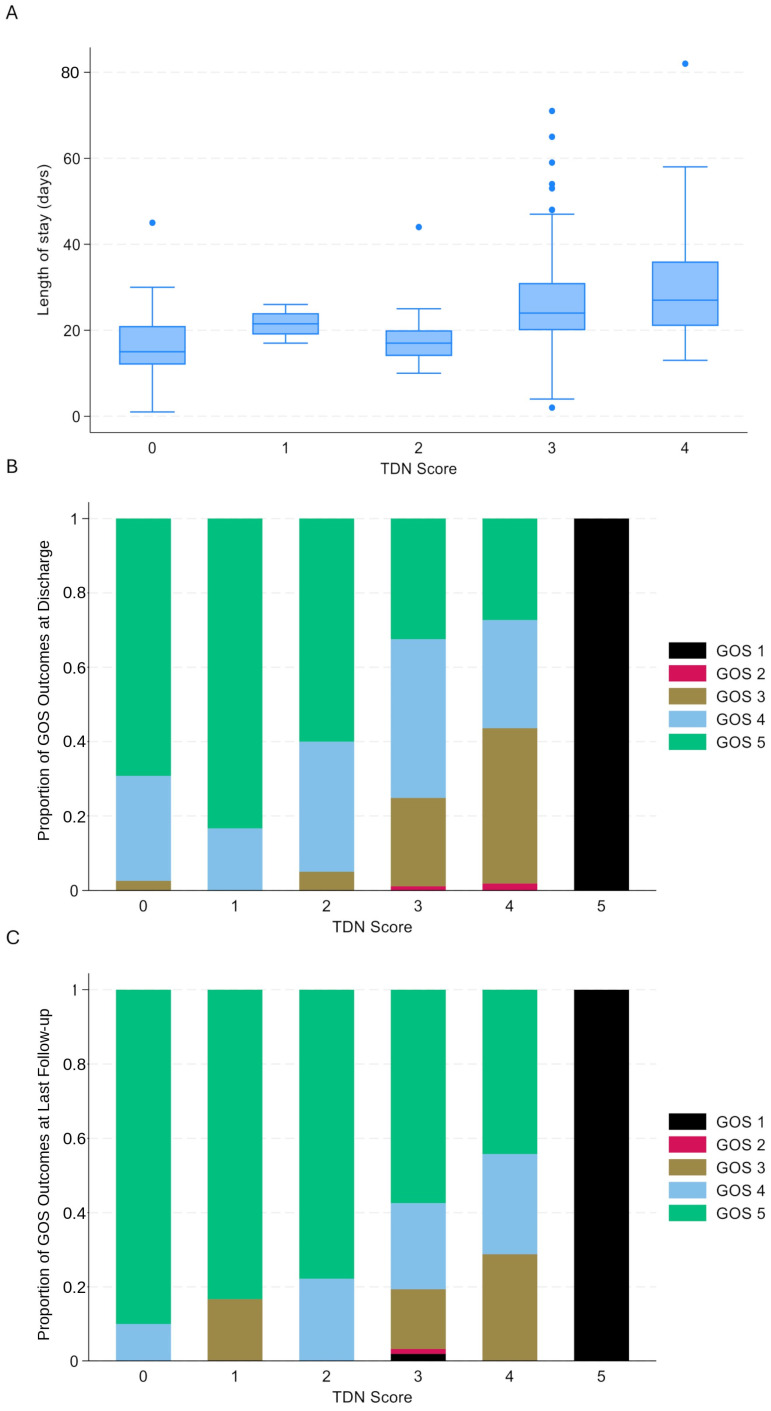
(**A**) Box plot showing the correlation between TDN grade and length of hospital stay (LOS). A moderate positive correlation (rho = 0.4, *p* < 0.001) demonstrates increasing hospital stay with higher TDN grades. (**B**) Stacked column chart showing Glasgow Outcome Scale (GOS) distribution at discharge by TDN grade. Higher TDN grades are associated with worse functional outcomes (rho = −0.56, *p* < 0.0001). (**C**) Stacked column chart showing Glasgow Outcome Scale (GOS) distribution at last follow-up by TDN grade. The correlation remains strong at long-term follow-up (rho = −0.58, *p* < 0.0001).

Mean LOS in days was 23.6 (SD 11.8). Length of stay increased by +7.63 days (95% CI 3.67–11.60) with the occurrence of an AE (*p* < 0.001). In the length of stay analysis, the TDN grade showed a significant positive, moderate correlation with LOS (rho = 0.4, 95% CI 0.31–0.50, *p* < 0.001, Figure 1A). We additionally performed a sensitivity analysis including all patients, assigning deaths (TDN = 5) a LOS value at the upper extreme of the observed distribution, such that they received the highest ranks in the Spearman correlation. This sensitivity analysis yielded a higher correlation (Spearman’s rho = 0.64, 95% CI 0.57–0.69, *p* < 0.001). However, because deceased patients are by definition at the top of the TDN scale, assigning them the highest LOS ranks mechanically inflates the rank correlation. The true correlation likely lies between the primary (rho = 0.4) and sensitivity (rho = 0.64) estimates.

### 3.2. TDN Grade and Functional Outcomes

The TDN grade assessed during the index hospitalization demonstrated strong associations with functional outcomes across all time points. At discharge, TDN was negatively correlated with GOS (rho = −0.56, 95% CI −0.62 to −0.49, *p* < 0.0001, Figure 1B). This negative correlation persisted and strengthened at last follow-up, with GOS showing a strong negative correlation (rho = −0.58, 95% CI −0.65 to −0.5, *p* < 0.0001, Figure 1C). The stacked column charts demonstrate the progressive worsening of functional outcomes with increasing TDN grades.

### 3.3. Discrimination of the TDN Grade for Poor Outcomes

**Figure 2:**
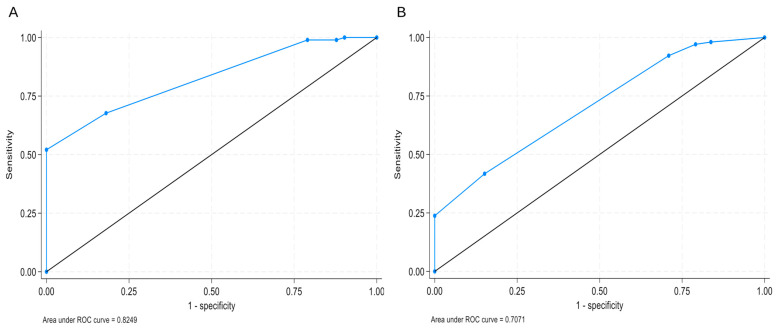
(**A**) Receiver operating characteristic (ROC) curve for TDN grade predicting unfavorable outcome (GOS 1–3) at last follow-up. The area under the curve (AUC) is 0.82, indicating good discrimination. (**B**) Receiver operating characteristic (ROC) curve for TDN grade predicting employment status at last follow-up (excluding patients above retirement age). The AUC is 0.71, indicating good discrimination for return-to-work capacity.

The TDN grade demonstrated a good predictive value for unfavorable outcomes at last follow-up. For poor GOS at last follow-up (defined as GOS 1–3), the TDN grade achieved an area under the ROC curve (AUC) of 0.82 (Figure 2A).

### 3.4. TDN Grade and Employment Status

Information on employability at last follow-up was available for 190 patients below retirement age. The TDN grade showed good discrimination for employment status with an AUC of 0.71 (Figure 2B).

### 3.5. TDN Grade and Correlations with Initial SAH Severity Scores

Analysis of initial SAH severity scores revealed significant positive correlations with TDN. The strongest correlations were observed with the Hunt & Hess scale (rho = 0.43, 95% CI 0.34–0.51, *p* < 0.0001) and WFNS score (rho = 0.42, 95% CI 0.33–0.50, *p* < 0.0001), followed by Fisher Score (rho = 0.27, 95% CI 0.17–0.36, *p* < 0.0001) and BNI Score (rho = 0.18, 95% CI 0.08–0.28, *p* = 0.0009, see Table 4).

## 4. Discussion

Our analysis revealed several key findings regarding the predictive value of the TDN grade in aSAH patients. This study represents the first application of the TDN grade beyond perioperative neurosurgical contexts, extending its use to a complex cerebrovascular population and demonstrating its potential as a unified measure of both procedure- and disease-related complication burden. Most importantly, it demonstrated significant correlations with length of hospital stay and functional outcomes at discharge and last follow-up, with good discrimination for poor outcomes.

The 89.6% AE rate in our cohort is substantially higher than rates reported in general neurosurgical cohorts but consistent with the aSAH-specific literature, which reports complication rates of 79% [20], and when disease-related events are included, up to 100% [21]. This reflects the deliberately inclusive AE definition we applied—capturing both intervention-related and disease-related complications—rather than a unique safety profile of our institution. This comprehensive scope is precisely what the TDN grade is designed to handle in this population.

Length of stay in aSAH patients depends on multiple factors, including initial bleeding severity, complications, and institutional protocols [20,21,22]. The TDN grade showed a moderate correlation with LOS (rho = 0.4), suggesting that the comprehensive assessment of both intervention-related and disease-specific AEs provides valuable insight into hospitalization duration in aSAH. The finding that AEs prolonged hospital stay by 7.63 days highlights the substantial impact of AEs on resource utilization. Given that hospital stay represents a significant economic burden in aSAH patient care [23,24,25], the TDN grade’s ability to predict LOS could serve as a valuable tool for resource planning and quality improvement initiatives.

Our handling of deceased patients in the LOS analysis warrants careful interpretation. Excluding deceased patients from the primary analysis likely biases the correlation downward by removing the most severely affected cases. Conversely, the sensitivity analysis—assigning deceased patients the highest LOS ranks—imposes the assumption that they would have had the longest stays, mechanically inflating the rank correlation by placing them at the top of both variables. Neither estimate should be interpreted as unbiased; the true correlation likely lies between rho = 0.4 and rho = 0.64. A formal competing-risks analysis would address this limitation more rigorously and represents a direction for future work.

The strong negative correlation of the TDN grade with functional outcomes at discharge and last follow-up, as well as employability at last follow-up, reflects the comprehensive nature of this scoring system in capturing the complex interplay between treatment-related AEs and disease-specific factors such as vasospasm and delayed cerebral ischemia [26,27]. The good discrimination for unfavorable outcomes at last follow-up (AUC = 0.82) indicates the TDN grade’s effectiveness in identifying patients at risk of poor long-term outcomes, making it a valuable prognostic tool for clinical decision-making and patient counseling.

The moderate to good discrimination for employment status (AUC = 0.71) represents a particularly clinically relevant finding, as return to work is an important quality of life indicator for aSAH survivors. This finding extends the value of the TDN grade beyond traditional outcome scales, demonstrating its relevance in predicting broader aspects of recovery and reintegration into daily life.

The correlation between initial SAH severity scores and the TDN grade provides important insights into the relationship between initial presentation and subsequent AEs. Our findings demonstrated that the TDN grade showed moderate to strong correlations with established clinical grading systems, particularly the Hunt & Hess scale and WFNS score. This relationship suggests that patients presenting with more severe initial symptoms are more likely to experience adverse events during their clinical course [27]. Although the abovementioned SAH grading systems are valuable in predicting the occurrence of some AEs (e.g., vasospasms) and outcomes following aSAH, as has been shown previously [28,29], AEs that may be unpredictable can accumulate over the post-hemorrhagic course and substantially modify prognosis. The TDN grade addresses this gap by providing a dynamic, standardized framework to quantify the overall burden of complications, both intervention- and disease-specific, throughout the patient’s clinical course. By integrating this longitudinal perspective, the TDN grade complements traditional admission scores and fulfills an unmet need for comprehensive complication assessment in aSAH.

In this context, this study represents the first validation of the TDN grade in a complex cerebrovascular disease that includes both neurosurgical and neuroendovascular interventions, extending its application beyond its original intended scope. However, unlike other neurosurgical conditions, aSAH involves complex interactions between the initial hemorrhage and secondary brain injury mechanisms (such as early brain injury, delayed cerebral ischemia, and spreading depolarizations), subtleties that may not be adequately reflected in a broad grading system originally designed for general neurosurgical procedures. Future refinements of the TDN classification for aSAH could include, or be used alongside, tools integrating disease- and patient-specific prognostic factors, such as initial hemorrhage severity, aneurysm characteristics, age, or comorbidities, to further enhance predictive precision.

A further consideration is the retrospective application of the TDN grade. Although core outcome and AE data were collected prospectively, the TDN grade itself was applied retrospectively to documented events. This may introduce information bias if AE documentation was incomplete, most likely toward underestimation of TDN scores in less severe cases. The inter-rater reliability of the TDN grade has been demonstrated previously [9], partially mitigating this concern.

Several considerations underpin our multivariable model. We selected GOS ≤ 3 at last follow-up as the outcome because it represents the most clinically meaningful endpoint for aSAH survivors and is widely used in the field. The predictor set (age, sex, WFNS, TDN) was chosen to adjust for the strongest established prognostic factors while keeping the model parsimonious; Hunt & Hess and Fisher scores were not included due to high collinearity with WFNS. With approximately 96 poor outcomes among 301 complete cases, the events-per-variable ratio (~24) substantially exceeds the conventional threshold of 10, supporting the statistical adequacy of the model.

A conceptual concern is that the TDN grade incorporates a disability component (mRS resulting from AEs), which shares conceptual ground with the GOS outcome and could introduce circularity. We note, however, that the mRS within TDN is assessed at the time of each AE during the index hospitalization, whereas GOS at follow-up reflects functional status months later, after rehabilitation, recovery, and potential late deterioration. The strong but imperfect association (TDN OR 5.76 for poor GOS) is therefore consistent with TDN capturing acute complication burden that influences but does not deterministically predict long-term outcome. Nonetheless, future analyses examining each TDN dimension separately, or using outcomes less overlapping with disability (e.g., employment status, where we observed an AUC of 0.71), would help disentangle this question.

Several other limitations warrant discussion. The single-center nature of our study may limit generalizability, particularly given the heterogeneity in aSAH management protocols across institutions. Also, follow-up timing varied considerably, potentially affecting our long-term outcome assessments and introducing selection bias. Furthermore, our assessment cannot be extrapolated to all aSAH patients as we excluded conservatively managed patients (9.6%), which likely resulted in underestimation of overall mortality and morbidity, as most of these patients had palliative care approaches. This exclusion creates a selection bias toward patients deemed suitable for intervention, which likely overestimates the TDN grade’s discriminative performance in the broader aSAH population. Notably, structured baseline data for excluded patients (*n* = 70) were not systematically collected, precluding a direct comparison of included and excluded cohorts and limiting our ability to formally quantify selection bias. This represents an important source of residual selection bias that cannot be quantitatively excluded. Additionally, severely disabled patients may have been less likely to attend follow-up appointments, potentially biasing our long-term outcome data toward better outcomes. For employment analysis, we excluded patients above retirement age, which may limit the generalizability of these findings and potentially overestimate the score’s discriminative ability for return to work.

## 5. Conclusions

Our study suggests the validity of the TDN grade as a useful predictor of length of hospital stay among in-hospital survivors and of functional outcomes in aSAH patients. Good discrimination for unfavorable outcomes (AUC = 0.82) and for employment status (AUC = 0.71) supports its potential utility as a comprehensive prognostic tool that captures both intervention-related and disease-specific AEs, with potential value for clinical practice, patient counseling, and resource planning. However, assessment of AEs alone is not sufficient, and a comprehensive approach that integrates disease-specific factors along with complication grading is necessary to achieve optimal prognostic accuracy in this complex patient population. Given the single-center retrospective design and inherent selection biases, prospective multicenter validation is required before broader clinical adoption, ideally integrating the TDN grade with existing outcome prediction tools.

## Figures and Tables

**Table 1:** Characteristics of the cohort.

Variable	Overall Cohort (*n* = 355)	Surgical Aneurysm Treatment (*n* = 158)	Endovascular Aneurysm Treatment (*n* = 197)	*p*-Value
Age y, (mean, SD)	57.2 (12.9)	56.0 (12.1)	58.0 (13.5)	0.36
Female sex, *n* (%)	235 (66.2)	101 (63.9)	134 (68)	0.38
Smoker, *n* (%)	91 (25.6)	35 (22.2)	56 (28.4)	0.3
Medical History				
- Hypertension, *n* (%)	91 (25.6)	34 (21.5)	57 (28.9)	0.06
- Diabetes Mellitus, *n* (%)	14 (3.9)	7 (4.4)	7 (3.6)	0.58
- On statin medication, *n* (%)	21 (5.9)	7 (4.4)	14 (7.1)	0.36
- Aspirin, *n* (%)	31 (8.7)	10 (6.3)	21 (10.7)	0.27
Aneurysm location, *n* (%)				<0.01 *
- Anterior circulation	252 (71.0)	124 (78.5)	128 (65.0)	
- Posterior circulation	104 (29.3)	35 (22.2)	69 (35.0)	
Mean Aneurysm size mm (SD)	7.4 (4.5)	7.3 (4.2)	7.9 (5.3)	0.30
Multiple aneurysms, *n* (%)	94 (26.5)	46 (29.1)	48 (24.4)	0.60
Rebleeding, *n* (%)	22 (6.2)	8 (5.1)	14 (7.1)	0.52
Craniectomy, *n* (%)	35 (9.9)	21 (13.3)	14 (7.1)	0.048
EVD insertion, *n* (%)	246 (69.3)	94 (59.5)	152 (77.2)	<0.01 *
Shunt dependency, *n* (%)	127 (35.8)	54 (34.2)	73 (37.1)	0.62
TDN grade, *n* (%)				0.34 ^1^
- 0	39 (11.3)	18 (11.4)	21 (10.7)	
- 1	6 (1.7)	3 (2)	3 (1.6)	
- 2	18 (5.2)	10 (6.7)	8 (4.3)	
- 3	184 (53.5)	80 (50.6)	104 (52.5)	
- 4	49 (14.2)	23 (14.6)	26 (13.2)	
- 5	49 (14.2)	18 (11.4)	31 (15.6)	
LOS d, mean (SD)	23.6 (11.8)	24.5 (11.6)	23.2 (12.6)	0.32
Mortality during hospitalization, *n* (%)	51 (14.4)	19 (12)	32 (16.2)	0.29

EVD = extraventricular drainage; LOS = length of hospital stay; *n* = Number; TDN = Therapy–Disability–Neurology. In case of missing values in the predictors, the number is displayed as a fraction. ^1^ Patients who received both treatment modalities or bypass surgery were excluded from the analysis (*n* = 11). * Statistically significant (*p* < 0.05).

**Table 2:** Risk factors for AE development (univariate logistic regression).

Variable	Odds Ratio (95% CI)	*p*-Value
Sex	0.95 (0.46–1.97)	0.898
Age (per year)	1.04 (1.01–1.07)	0.004
WFNS (per point)	2.973 (1.870–4.726)	<0.001
Hunt & Hess (per point)	2.961 (1.950–4.499)	<0.001
Fisher Score (per point)	4.593 (2.512–8.395)	<0.001
BNI Score (per point)	2.564 (1.619–4.059)	<0.001
Diabetes Mellitus	1.655 (0.758–3.614)	0.218
Hypertension	1.885 (0.760–4.676)	0.172

**Table 3:** Multivariable logistic regression for unfavorable outcome (GOS ≤ 3) at last follow-up.

Variable	Odds Ratio (95% CI)	*p*-Value
Age (per year)	1.03 (1.01–1.06)	0.019
Sex (female)	0.84 (0.42–1.68)	0.620
WFNS (per point)	1.40 (1.14–1.73)	0.001
TDN grade (per point)	5.76 (3.69–9.01)	<0.001

Complete-case analysis (*n* = 301 of 355 patients with available GOS at last follow-up).

**Table 4:** Correlation of TDN with outcome parameters and initial SAH severity scores.

Variable	N	TDN Overall rho	TDN Overall *p*-Value
Length of Stay ^1^	305	0.4	<0.0001
WFNS	355	0.421	<0.0001
Hunt & Hess Score	355	0.435	<0.0001
Fisher Score	355	0.269	<0.0001
BNI Score	355	0.176	0.0009
KPS at Discharge	355	−0.572	<0.0001
GOS at Discharge	355	−0.559	<0.0001
KPS at Last Follow-up	250	−0.575	<0.0001
GOS at Last Follow-up	250	−0.582	<0.0001

^1^ Length of stay analysis excludes deaths (TDN 5).

## Data Availability

The datasets generated and/or analyzed during the current study are not publicly available due to patient privacy and data protection regulations, but are available from the corresponding author on reasonable request.

## Supplementary Materials

The following supporting information can be downloaded at https://www.mdpi.com/article/10.3390/brainsci16060599/s1; Figure S1: Study flowchart; Table S1: Most common adverse events (AEs) in our cohort.

## Abbreviations

The following abbreviations are used in this manuscript:

AE	Adverse Event
SAH	Aneurysmal Subarachnoid Hemorrhage
AUC	Area Under the Curve
AUROC	Area Under the Receiver Operating Characteristic Curve
BNI	Barrow Neurological Institute
CI	Confidence Interval
CTA	Computed Tomography Angiography
DSA	Digital Subtraction Angiography
EVD	Extraventricular Drainage
GOS	Glasgow Outcome Scale
ICP	Intracranial Pressure
IQR	Interquartile Range
KPS	Karnofsky Performance Status
LOS	Length of Stay (Hospital)
MRA	Magnetic Resonance Angiography
mRS	Modified Rankin Scale
OR	Odds Ratio
SD	Standard Deviation
STROBE	Strengthening the Reporting of Observational Studies in Epidemiology
TDN	Therapy–Disability–Neurology (Grade)
WFNS	World Federation of Neurological Surgeons (Score)
