# Supplementary material for: Assessment of Adverse Events Using the Therapy–Disability–Neurology (TDN) Grading System in a Cohort of Aneurysmal Subarachnoid Hemorrhage Patients: A Single-Center Retrospective Cohort Study
Source: Brain Sci. 2026 May 31;16(6):599. doi: 10.3390/brainsci16060599 (PMC13297518; doi:10.3390/brainsci16060599)
Supplement: Supplementary file 1 [file brainsci-16-00599-s001.zip › brainsci-4304759-supplementary-revised.pdf]

**Supplemental Material:**

**Assessment of adverse events using the Therapy-Disability-Neurology (TDN) Grading-System in a cohort of Aneurysmal Subarachnoid Hemorrhage Patients**

Vincens Kälin, Alexis Paul Romain Terrapon, Anna Maria Zeitlberger, Gareth Ambler, Svenja Maschke, Ahmed El-Garci, Sara Bonasia, Oliver Bozinov, Marian Christoph Neidert, Isabel Charlotte Hostettler

**Corresponding author:**

Dr. Vincens Kälin, Department of Neurosurgery, HOCH Health Ostschweiz, Cantonal Hospital St. Gallen, Rorschacher Strasse 95, 9007 St. Gallen, Switzerland; Phone: +41 71 494 60 21, email: Vincens.kaelin@gmail.com

**Supplemental Figure S1:** Study Flowchart

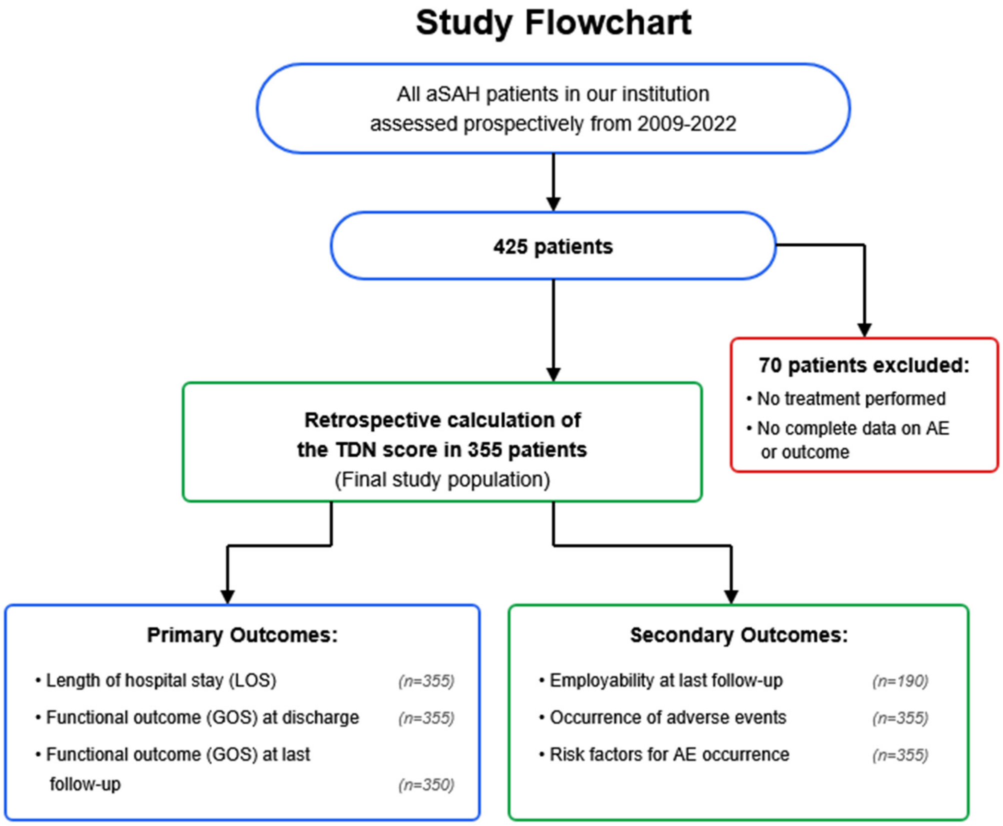

**Supplemental Table S1:** Most common adverse events (AEs) in our cohort

| Adverse event | Count | Percentage |
|---|---|---|
| Hydrocephalus | 222 | 70.0% |
| Cerebral vasospasm | 110 | 34.7% |
| Pneumonia | 38 | 12.0% |
| Elevated intracranial pressure /brain edema | 37 | 11.7% |
| Infarction | 24 | 7.6% |
| Ventriculitis/Meningitis | 24 | 7.6% |
| Urinary tract infection | 22 | 6.9% |
| Rebleeding/Rerupture | 14 | 4.4% |
| Electrolyte disorders | 12 | 3.8% |
| Infection (general) | 9 | 2.8% |
| Thrombosis/Embolism | 8 | 2.5% |
| Delirium | 6 | 1.9% |
| Cardiac complications | 6 | 1.9% |
| Epilepsy | 3 | 0.9% |
| Sepsis | 2 | 0.6% |
| Other adverse events | 51 | <1% each |
